# *Hylocereus polyrhizus* Peel Extract Retards Alcoholic Liver Disease Progression by Modulating Oxidative Stress and Inflammatory Responses in C57BL/6 Mice

**DOI:** 10.3390/nu12123884

**Published:** 2020-12-18

**Authors:** Wan-Ju Yeh, Chia-Chun Tsai, Jung Ko, Hsin-Yi Yang

**Affiliations:** 1 Graduate Program of Nutrition Science, National Taiwan Normal University, Taipei 106, Taiwan; wandayeh@ntnu.edu.tw; 2 School of Nutrition and Health Sciences, Taipei Medical University, Taipei 110, Taiwan; jessie4f@gmail.com; 3 Department of Applied Biological Chemistry, Graduate School of Agricultural and Life Sciences, The University of Tokyo, Tokyo 113-3584, Japan; a532732002@hotmail.com; 4 Department of Nutritional Science, Fu Jen Catholic University, New Taipei City 24205, Taiwan

**Keywords:** alcoholic liver disease, red pitaya, fatty liver, oxidative stress, inflammation

## Abstract

Alcoholic liver disease (ALD) has become a health problem as alcohol consumption has increased annually. Hepatic lipid accumulation, oxidative stress, and inflammation are important factors in the progression of ALD. Red pitaya (*Hylocereus polyrhizus* (Weber) Britt. & Rose) peel is rich in polyphenols and betanins, which possess antioxidative and anti-inflammatory properties. Therefore, the aim of this study was to investigate the effects of red pitaya peel extract (PPE) on ALD and explore the associated mechanisms. C57BL/6 J mice were administered an ethanol liquid diet for 11 weeks with or without two different doses of PPE (500 and 1000 mg/kg BW). PPE treatment significantly ameliorated liver injury and hepatic fat accumulation, and it improved hepatic lipid metabolism via increases in AMPK and PPAR-α protein expression and a decrease in SREBP-1 expression. In addition, PPE inhibited CYP2E1 and Nrf2 protein expression, reduced endotoxin levels in the serum, and decreased TLR4 and MyD88 expression and inflammatory cytokine TNF-α and IL-1β levels in the liver. In conclusion, these findings suggest that PPE may prevent the progression of ALD by modulating lipid metabolism and reducing oxidative stress and inflammatory responses.

## 1. Introduction

According to a report by the World Health Organization, alcohol consumption is related to the risks of many diseases and public health problems [1]. Alcohol consumption may lead to alcoholic liver disease (ALD), which is the most prevalent chronic liver disease in the world and can progress from fatty liver to steatohepatitis and even fibrosis and cirrhosis [2]. Alcohol-induced lipid metabolism impairment, increased oxidative stress, and proinflammatory responses play important roles in the pathogenesis of ALD. Proper therapeutic management at an early stage of disease may be helpful to retard disease progression or ameliorate liver injury [3].

Long-term alcohol exposure may lead to ectopic fat accumulation in the liver because of an imbalance in lipid metabolism regulated through AMP-activated protein kinase (AMPK) [4]. The inhibitory effect of ethanol on AMPK phosphorylation may upregulate hepatic sterol regulatory element-binding protein 1c (SREBP-1c) expression to increase fatty acid synthesis [5] and downregulate peroxisome proliferator-activated receptor (PPAR)-α to decrease fatty acid oxidation [6].

The activation of cytochrome P450 2E1 (CYP2E1) caused by ethanol metabolism increases oxidative stress [7] and stimulates the activation of nuclear factor kappa-light-chain-enhancer of activated B cells (NF-κB) to induce secretion of proinflammatory cytokines, such as tumor necrosis factor (TNF)-α and interleukin (IL)-1β [8]. In addition, recent studies have reported that alcohol consumption also stimulates the progression of liver disease through the gut–liver axis [9,10]. Chronic ethanol exposure is related to dysbiosis and gut leakiness in vivo [10], and an increase in circulating endotoxin levels may lead to hepatic inflammation via activation of the Toll-like receptor 4 (TLR4)–myeloid differentiation primary response gene 88 (MyD88) signaling pathway [11].

In addition to medical treatment and lifestyle modification, food components with antioxidative or anti-inflammatory potentials may also have ameliorative effects on the progression of ALD [3]. Red pitaya (*Hylocereus polyrhizus* (Weber) Britt. & Rose) is one of the most popular fruits in Taiwan and has shown many health-promoting bioactivities such as antioxidant, anti-inflammatory, and antiangiogenic properties [12]. It also shows beneficial effects on hepatic steatosis and hepatic injury in diet-induced obese animal models [13]. Red pitaya peel represents almost one-third of the total fruit weight; it contains more phenolic compounds and betanins, the most abundant betacyanin, and shows better antioxidative effects than the fruit flesh [14,15]. Betanin, the main red pigment in pitaya peel, was reported to moderate lipid metabolism and decrease lipid peroxidation in mice fed a high-fat diet [16,17]. Therefore, the aim of this study was to investigate the effects of red pitaya peel extract (PPE) on ALD and clarify the possible underlying mechanisms.

## 2. Materials and Methods

### 2.1. PPE Preparation

Fresh red pitaya peel (Da-Hong, Kaohsiung, Taiwan) was cut into small pieces, freeze-dried, and ground into powder, and the recovery rate was 10.5 ± 1.2%. The extraction of pitaya peel followed a previously reported method [18]. Briefly, the peel powder was extracted with 50% ethanol (1:15 (*w*/*v*), room temperature) for 30 min with a magnetic stirrer (300 rpm) and was centrifuged at 2400× *g* at 4 °C for 15 min. The supernatant was then filtered (Adventec No. 1, Tokyo, Japan), and the filtrate was concentrated by vacuum evaporation at temperatures not exceeding 38 °C in the dark. The concentrate was freeze-dried, ground into powder, and stored protected from light at −20 °C until further use. The total polyphenol content of PPE was determined as described previously [19] and is expressed as mg gallic acid equivalents. Betacyanin levels in PPE were measured spectrophotometrically according to the method published by Cai et al. [20] at 538 nm, which is the maximum absorbance of betacyanins. The total betacyanin content was then calculated with equation (1). In addition, the total antioxidant capacity was detected with a ferric reducing antioxidant power (FRAP) assay using a commercial kit (STA-859, Cell Biolabs, San Diego, CA, USA) and is expressed as ascorbic acid equivalents.

total betacyanin content (mg/g) = ((A × DF × MW × V/εLW))(1)

where A is the absorbance value at 538 nm, DF is the dilution factor, MW is the molecular weight of betanin (550 g/mol), V is the total volume of the sample solution (mL), ε is the mean molar absorptivity (60,000 L/mol/cm in H_2_O), L is the path length (1 cm), and W is the sample weight in the sample solution (g).

### 2.2. Experimental Design

Forty 7-week-old male C57BL/6 mice were purchased from the National Laboratory Animal Breeding and Research Center (Taipei, Taiwan). The experimental protocols were reviewed by the Institutional Animal Care and Use Committee of Taipei Medical University, and investigators followed protocols described in the “Guide for the Care and Use of Laboratory Animals”. All protocols were approved by the Institutional Animal Care and Use Committee (LAC-2015-0235). Mice were maintained in a room at 22 ± 2 °C with 55% ± 5% humidity and a 12-h light/dark cycle and fed a Lieber–DeCarli ethanol liquid diet to induce ALD [21]. Mice were randomly assigned to 4 groups (*n* = 10) for an 11-week experiment after a 1-week adaptation period. The diet composition is shown in Table 1. The C group was fed a control liquid diet, while the E group was pair-fed an isocaloric ethanol liquid diet, and the E + LP and E + HP groups were fed an ethanol liquid diet with 500 or 1000 mg/kg body weight (BW) PPE, respectively. Body weight was recorded every week, and food intake was recorded daily. At the end of the experimental period, mice were anesthetized with isoflurane and sacrificed, and we collected the blood, liver, and small intestine for further analysis.

The experimental liquid diet was prepared according to the previously reported modified Lieber–DeCarli ethanol liquid diet, and the control liquid diet used isocaloric maltodextrin substituted for ethanol. Casein (high N), L-cystine, choline bitartrate, cellulose (non-nutritive bulk), mineral mixture (AIN-93M), and vitamin mixture (AIN-93M) were obtained from ICN Biochemicals (Aurora, OH, USA). Dextrin–maltose was obtained from Corn Products International, Inc. Soybean oil was obtained from Taiwan Sugar Corporation. Xanthan gum and ethanol were obtained from Sigma (St. Louis, MO, USA).

### 2.3. Blood Sample Collection and Analysis

Blood samples were collected from the inferior vena cava into tubes and centrifuged, and the serum was stored at −80 °C until analysis. Serum triglycerides (TGs), alanine aminotransferase (ALT), and aspartate aminotransferase (AST) were analyzed using a Roche Modular P800 autoanalyzer (Roche Diagnostics, Indianapolis, IN, USA). Serum γ-glutamyl transpeptidase (γ-GT) activity was measured spectrophotometrically using a commercial kit (Randox GT2750, Antrim, UK). Serum endotoxin levels were determined using the limulus amebocyte lysate (LAL) method (L00350, GenScript, Piscataway, NJ, USA).

### 2.4. Liver Collection and Analysis

Livers were weighed, and liver index was calculated as liver weight/body weight × 100. Liver lipids were extracted by the Folch method [22], and total cholesterol and TG concentrations in the liver were determined using diagnostic kits (Randox Laboratories, Antrim, UK). To analyze hepatic lipid peroxidation and proinflammatory cytokine levels, liver tissues were homogenized with a buffer (50 mM Tris–HCl, 150 mM NaCl, 1% NP-40, 0.1% SDS) containing a protease inhibitor (Roche, Mannheim, Germany) and centrifuged (3000× *g*, 4 °C, 15 min), and the supernatant was used to determine the malondialdehyde (MDA) [23], TNF-α and IL-1β concentrations (R&D systems, Minneapolis, MN, USA). To measure the glutathione (GSH) and ROS concentration, liver tissues were homogenized with PBS (pH 7.4) containing 1 mM ethylenediaminetetraacetic acid and centrifuged (10,000× *g*, 4 °C, 15min), and the supernatant was used to determine the GSH level after deproteinization and was also subjected to a ROS/RNS assay. GSH (703,002, Cayman Chemical, Ann Arbor, MI, USA) and ROS/RNS (STA-347, Cell Biolabs, San Diego, CA, USA) were determined with commercial kits following the instructions provided by the manufacturers.

To analyze hepatic protein expression by Western blotting, we homogenized liver tissues with RIPA buffer containing 50 mM Tris-HCl, 150 mM NaCl, 1% NP-40, 0.1% SDS and a protease inhibitor and centrifuged the tissues at 4 °C and 10,000× *g* for 10 min. The protein concentration in the supernatant was measured with a Bio-Rad protein assay dye (Bio-Rad Laboratories, Hercules, 500-0002, CA, USA), and 30 g protein was separated on a sodium dodecyl sulfate (SDS)–polyacrylamide gel and transferred to a polyvinylidene difluoride (PVDF) membrane. In addition, nuclear and cytosolic fractions were extracted using commercial kits (BioVision, Milpitas, CA, USA) for analysis of Nrf2 and pNF-κB protein expression. Nonspecific binding sites were blocked by membrane incubation overnight at 4 °C in blocking buffer (Visual Protein, Taipei, Taiwan). After washing with PBS/Tween-20, the membranes were incubated with an anti-AMPK antibody (2532, Cell Signaling Technology, Danvers, MA, USA), anti-SREBP-1 antibody (GTX79299, GeneTex, Irvine, CA, USA), anti-PPAR-α antibody (Abcam, Cambridge, UK), anti-CYP2E1 antibody (Abcam, Cambridge, UK), anti-Nrf2 antibody (16396-1-AP, Proteintech, Rosemont, IL, USA), anti-TLR4 antibody (IMG-5031A, Imgenex, Centennial, CO, USA), anti-MyD88 antibody (Cell Signaling Technology, Danvers, MA, USA), anti-phosphor-NF-κB p65 (Ser536) antibody (Cell Signaling Technology, Danvers, MA, USA), anti-GAPDH antibody (BioLegend, San Diego, CA, USA), or anti-PCNA antibody (BioLegend, San Diego, CA, USA) at room temperature for 2 h, followed by incubation with a horseradish peroxidase (HRP)-gated secondary antibody (Jackson ImmunoResearch, West Grove, PA, USA). Then, the membranes were washed with buffer and treated with a chemiluminescence detection system (ECL, PerkinElmer, Waltham, MA, USA) to develop the immune complexes. The BioSpectrum AC image system, UVP Visionwork LS software, and Image-Pro Plus 4.5 (Media Cybernetic, Rockville, MD, USA) were used to quantify the bands. Equal loading of the total protein was verified using a commercially available antibody against GAPDH or PCNA, and the results are expressed as the ratio of the target protein level to the control protein level.

### 2.5. Histopathological Analysis

Liver tissues perfused with 0.9% NaCl were fixed in 10% (*v*/*v*) formaldehyde and then stained with hematoxylin and eosin (H&E) for histopathological analysis. Morphological changes were examined on a blinded basis by a pathologist, and hepatic steatosis and inflammation were scored according to a previously reported scoring system [24].

### 2.6. Absorption Test

To clarify whether PPE can directly affect ethanol absorption, we purchased twenty 8-week-old male Wistar rats from BioLASCO (Taipei, Taiwan) and randomly assigned them to two groups (*n* = 10), followed by a 1-week adaptation period. After a 12-h fasting period, we gave the ethanol control group (EE) a single dose of ethanol (30% (*v*/*v*), 1 mL/kg BW) via oral gavage and gave the experimental group (EP) a single dose of ethanol plus 1000 mg/kg BW PPE. We collected blood samples from the tail vein at 0, 30, 60, 120, and 180 min; determined the plasma ethanol concentration (MAK076, Sigma-Aldrich, St. Louis, MO, USA); and calculated the area under the curve (AUC).

### 2.7. Statistical Analysis

Results are expressed as the mean and standard deviation (SD) and were analyzed with the SAS program (v. 9.3; Cary, NC, USA). One-way analysis of variance (ANOVA) and Duncan’s multiple range test were used to analyze data among groups at the end of the study. Differences between two groups measured with the absorption test were analyzed using Student’s *t*-test. A *p*-value <0.05 was defined as the level of statistical significance.

## 3. Results

### 3.1. BW and Food Intake

The recovery rate of PPE from dried red pitaya peel powder was 15.2 ± 0.9%. The total polyphenol and total betacyanin contents of PPE were 33.6 ± 2.4 mg gallic acid equivalents/g and 6.3 ± 0.15 mg betanin equivalents/g, respectively, and the value of the FRAP assay was 21.6 mg ascorbic acid equivalents/g. We found no difference in BW or food intake among all groups during the 11-week experimental period, and there was also no difference in daily ethanol intake among the E, E + LP, and E + HP groups (15.6 ± 1.0, 14.9 ± 0.6, and 15.3 ± 0.5 g/kg/d; *p* > 0.05). In addition, the actual PPE intake in groups A and B was 500.1 ± 6.6 and 1019.5 ± 10.8 mg/kg/d.

### 3.2. Hepatic Injury

After consuming an ethanol liquid diet for 11 weeks, the E group had higher serum AST, ALT, and γ-GT activities than the C group, while both the E + LP and E + HP groups had lower AST, ALT, and γ-GT activities than the E group (Table 2).

### 3.3. Lipid Metabolism

**Figure 1:**
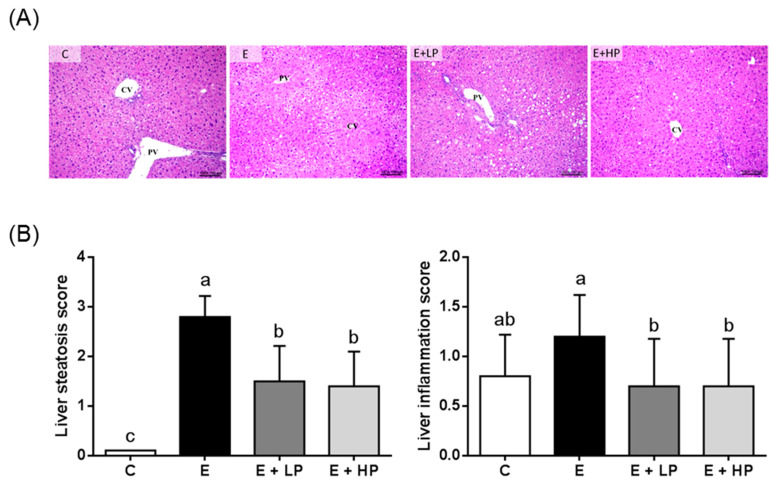
Histopathological changes in the liver of mice at the end of the study. (**A**) Photomicrographs of hematoxylin and eosin (H&E)-stained mouse liver sections (original magnification, 100×). (**B**) Pathological scores. Values are expressed as the mean ± SD (*n* = 10). Significant differences (*p* < 0.05) are identified by different letters (a,b). C, control group; E, ethanol group; E + LP, low-dose PPE group; E + HP, high-dose PPE group; CV, central vein; PV, portal vein.

Long-term consumption of an ethanol liquid diet led to elevations in the serum and hepatic TG concentrations in mice, and both the E + LP and E + HP groups had lower hepatic TG levels than the E group (Table 2). Histological evaluation of liver sections showed severe diffuse steatosis, focal cellular ballooning, and lobular inflammation in the E group, while steatosis was significantly decreased in the PPE-supplemented groups. Mild mononuclear cell infiltration was only found in the E group. In addition, the E + LP and E + HP groups also had lower steatosis and inflammation scores than the E group (Figure 1).

**Figure 2:**
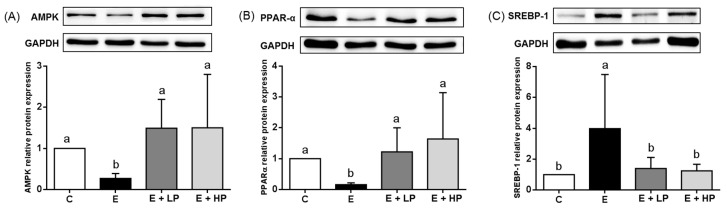
Hepatic (**A**) AMPK, (**B**) PPAR-α, and (**C**) SREBP-1 protein expression in mice at the end of the study. Values are expressed as the mean ± SD (*n* = 10). Significant differences among groups (*p* < 0.05) are identified by different letters (a,b). C, control group; E, ethanol group; E + LP, low-dose PPE group; E + HP, high-dose PPE group; AMPK, AMP-activated protein kinase; PPAR-α, peroxisome proliferator-activated receptor-α; SREBP-1, sterol regulatory element-binding protein-1.

We then analyzed lipid metabolism-related protein expression in the liver by Western blotting, and the results are shown in Figure 2. The E group had significantly lower AMPK and PPAR-α expression and higher SREBP-1 expression than the C group, while the E + LP and E + HP groups were not different from the C group.

### 3.4. Hepatic Oxidative Stress and Inflammation

**Figure 3:**
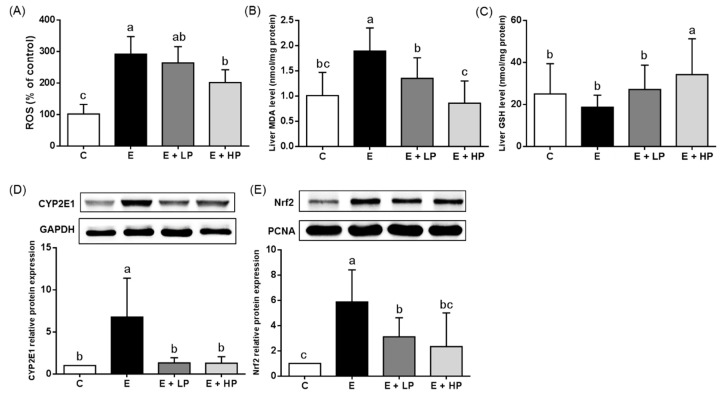
Hepatic (**A**) relative ROS level, (**B**) MDA and (**C**) GSH concentrations, and (**D**) CYP2E1 and (**E**) nuclear Nrf2 protein expression of mice at the end of the study. Values are expressed as the mean ± SD (*n* = 10). Significant differences (*p* < 0.05) are identified by different letters (a–c). C, control group; E, ethanol group; E + LP, low-dose PPE group; E + HP, high-dose PPE group; MDA, malondialdehyde; GSH, glutathione; CYP2E1, cytochrome P450 2E1; Nrf2, nuclear factor erythroid 2-related factor 2.

As shown in Figure 3, ethanol consumption significantly increased the relative level of ROS and the E + HP group had a significantly lower relative ROS level when compared to the E group. Ethanol also increased the lipid peroxidation marker MDA and produced a trend toward lower total GSH levels compared with control diet consumption. In addition, the E group also had higher hepatic CYP2E1 and Nrf2 protein expression. Both of the PPE-supplemented groups (E + LP and E + HP) had lower liver MDA concentrations and CYP2E1 and Nrf2 protein expression than the E group. Group B also had higher GSH levels than the E group.

**Figure 4:**
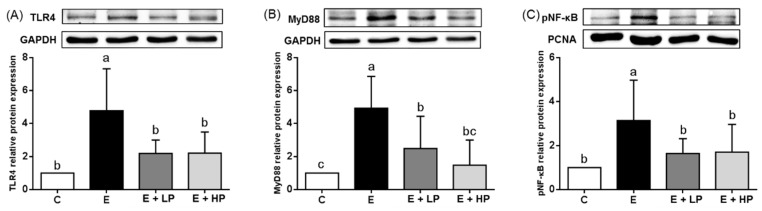
Hepatic (**A**) TLR4, (**B**) MyD88, and (**C**) nuclear pNF-κB protein expression of mice at the end of the study. Values are expressed as the mean ± SD (*n* = 10). Significant differences (*p* < 0.05) are identified by different letters (a–c). C, control group; E, ethanol group; E + LP, low-dose PPE group; E + HP, high-dose PPE group; TLR4, Toll-like receptor 4; MyD88, myeloid differentiation primary response gene 88; pNF-κB, phospho-nuclear factor kappa-light-chain-enhancer of activated B cells.

Because we found that groups A and B had lower inflammation scores in the histopathological analysis, we analyzed liver proinflammatory cytokine concentrations. As shown in Table 2, both PPE groups exhibited amelioration of the ethanol-induced elevations in hepatic TNF-α and IL-1β levels. To clarify the possible mechanisms, we further determined the serum endotoxin concentration and related hepatic protein expression. Ethanol consumption significantly elevated serum endotoxin levels. Both the E + LP and E + HP groups had lower endotoxin levels than the E group (Table 2). In addition, the E + LP and E + HP groups also had lower hepatic TLR-4, MyD88, and pNF-κB expression than the E group (Figure 4).

### 3.5. Absorption Test

The serum ethanol concentration reached a peak value at 60 min after oral gavage with ethanol or ethanol plus PPE, and no difference in the serum ethanol concentration at each time point or the AUC was found between the two groups (*p* > 0.05).

## 4. Discussion

Chronic exposure to alcohol induces and accelerates progression of ALD [25]. The Lieber–DeCarli (LDC) liquid diet is one of the most widely used experimental models of ALD in rodents for studying the pathogenesis of early stages of ALD [26]. In most studies, LDC diet induced various degrees of steatosis and inflammation without presenting fibrosis. During the pathogenesis of ALD, alcohol-induced fatty liver and hepatitis are reversible. In addition to alcohol cessation, medical therapy, and bioactive food components with the potential to modulate lipid metabolism, the antioxidative capacity and inflammatory responses play important roles and therefore have become therapeutic approaches applied in the early stage of ALD to retard disease progression [27]. Red pitaya peel is rich in bioactive phytochemicals [14] and was previously reported to ameliorate obesity and improve the antioxidative status in mice fed a high-fat diet [28]. To the best of our knowledge, this is the first study investigating the protective effects of PPE on alcohol-induced liver disease. In the present study, we fed mice a modified Lieber–DeCarli ethanol liquid diet mimicking chronic alcohol exposure in humans and found significant elevations in AST, ALT, and γ-GT activities at the end of the study in the ethanol liquid diet group compared to the isocaloric control group. In addition, histopathological analysis also identified steatosis in the E group. These results indicated the successful establishment of the ALD model. Many natural plant-derived bioactive components have been used in the treatment of ALD because of their antioxidative and anti-inflammatory activities [3]. Red pitaya betacyanins were previously shown to decrease high-fat diet-induced hepatic injury [28]. Our present study found that PPE could normalize ethanol-induced elevations in AST, ALT, and γ-GT activities at the dose of 500 mg/kg BW, which is equal to 41 mg/kg BW for humans (calculated based on body surface area) and showed the hepatoprotective potential of PPE in ALD.

Hepatic steatosis is the most common symptom that can be identified after the early stage of ALD. Alcohol consumption is related to impairments in de novo lipogenesis and fatty acid oxidation. Ethanol treatment in mice induces the expression of hepatic SREBP-1 and fatty acid synthesis related genes [29]. In addition, PPAR-α agonist treatment was shown to effectively ameliorate fatty liver and abnormal lipid metabolism in ethanol-fed mice by increasing the rate of β-oxidation [30]. In a diet-induced obesity model, pitaya-peel-derived betacyanins were found to induce fatty acid oxidation, decrease fatty acid biosynthesis by increasing adiponectin receptor 2 (*AdipoR2*) and carnitine palmitoyltransferase I (*Cpt1*) expression and decreasing fatty acid synthase (*Fas*) gene expression, and ameliorate hepatosteatosis [16]. In our study, we found that PPE supplementation reduced the hepatic lipid content. In addition, we also found that PPE groups had lower hepatic expression of AMPK, which plays a key role in regulating lipid metabolism and is associated with the downregulation of SREBP1 expression and upregulation of PPAR-α expression [31,32]. Consistently, we found lower SREBP1 expression and higher PPAR-α expression in both PPE groups. These results suggest that low and high dosages of PPE may both improve lipid metabolism and ameliorate hepatic fat accumulation by modulating the imbalance between lipid synthesis and oxidation.

Long-term and large amounts of alcohol consumption activate the expression of hepatic CYP2E1 and trigger oxidative stress, leading to tissue inflammation and injuries [7]. Lower GSH levels have been reported to be associated with ethanol-induced lipid peroxidation [33]. Nrf2 is an important regulator of adaptive antioxidative responses in vivo and is a therapeutic target in ethanol-induced oxidative injury [34]. Nrf2 activation may protect against ethanol-induced steatosis [35]. Studies have demonstrated that alcohol consumption upregulates Nrf2 expression for detoxification and, in contrast, supplementation with antioxidative compounds may normalize Nrf2 expression [36]. In addition, recent studies have also shown that Nrf2 overexpression may be implicated in the progression of ALD [37]. Red pitaya flesh and peel are rich in compounds with antioxidative and free radical scavenging potential [38]. In our study, we found that PPE inhibited ethanol-induced CYP2E1 and Nrf2 expression. We also found that PPE supplementation significantly decreased hepatic ROS production and MDA levels and increased GSH levels, especially in the high-dose group. A previous study reported that consuming betacyanins decreased circulating and hepatic MDA concentrations and increased the total antioxidative capacity by increasing hepatic GSH levels and glutathione peroxidase activity in mice fed a high-fat/high-cholesterol diet [17]. In a cell culture study, betanin showed free radical scavenging activity and reduced reactive oxygen species induced DNA damage [39]. A rodent study also indicated that betanin protects rats against paraquat-induced liver injury by elevating GSH levels and antioxidative enzyme activities [40]. These results suggest that one possible mechanism underlying the hepatoprotective effects of PPE supplementation during chronic ethanol exposure may be modulation of the antioxidative status in vivo.

Inflammation in ALD is caused by multiple pathobiological mechanisms. In addition to Kupffer cell activation and oxidative stress caused by ethanol metabolites, the gut–liver axis also plays an important role in the pathogenesis of ethanol-induced liver diseases [11]. Long-term alcohol consumption may disturb the normal gut microbiota distribution and tight junction protein expression, leading to increases in intestinal permeability and endotoxemia, which cause downstream NF-κB activation and proinflammatory cytokine secretion through upregulation of the TLR4–MyD88 pathway [10,41]. Studies using phytochemical intervention such as indole-3-carbinol showed protective effects against inflammatory liver injury by reducing oxidative stress, ameliorating gut leakiness, and reducing serum endotoxin [42]. In our study, we found a significant increase in circulating endotoxin levels, especially in the E + HP group, and TLR4 and MyD88 expression in our ethanol-fed mouse model, which was consistent with previous reports. It is worth noting that we also found lower hepatic TLR4, MyD88, and NF-κB protein expression in the PPE groups. Phenolic compounds, such as catechin, are reported to decrease inflammatory responses and endotoxemia induced by ethanol in rats [8]. In mice with diet-induced obesity, red pitaya-derived betacyanin treatment modulates the gut microbiota, including reducing the ratio of Firmicutes to Bacteroidetes and increasing the relative abundance of *Akkermansia* [27]. Dietary red pitaya juice also exhibits anti-inflammatory activities in mice with metabolic syndrome [43]. Therefore, PPE supplementation may also protect against ethanol-induced hepatic inflammation through the gut–liver axis.

## 5. Conclusions

**Figure 5:**
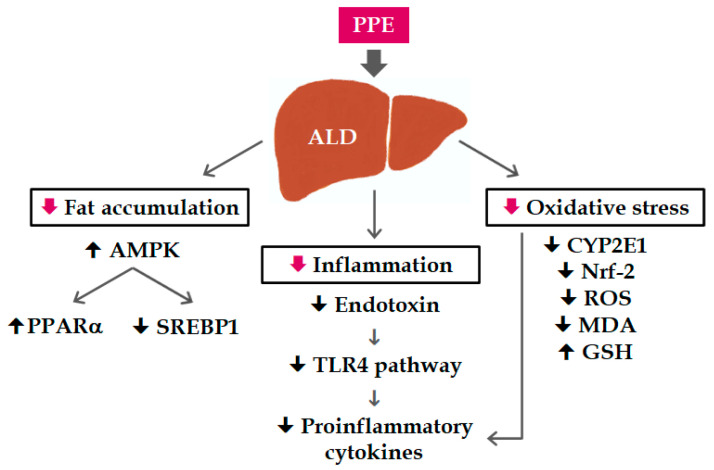
Possible mechanisms of the hepatoprotective effects of PPE on mice with alcoholic liver disease.

In conclusion, PPE supplementation can ameliorate hepatic steatosis and inflammation by regulating lipid metabolism and modulating oxidative stress and the hepatic TLR4–MyD88 pathway in ALD induced by chronic ethanol exposure (Figure 5). Red pitaya is a good source of dietary betacyanins, and its peel contains more bioactive compounds than its flesh. The results of this study may be used in dietary planning or functional food development.

## Figures and Tables

**Table 1:** Experimental liquid diet composition.

g/L	Control Liquid Diet	Ethanol Liquid Diet
Casein	41.4	41.4
L-Cystine	0.5	0.5
Soybean oil	48.5	48.5
Dextrin–maltose	98	35
Choline bitartrate	0.53	0.53
Cellulose	10	10
Xanthan gum	3	3
Vitamin mixture	2.2	2.2
Mineral mixture	7.7	7.7
Ethanol	0	36
Total energy (kcal)	996.1	996.1

**Table 2:** Serum and liver biochemical analysis of mice at the end of the study.

Variable	C	E	E + LP	E + HP
Serum				
AST (IU/L)	56.3 ± 7.1 ^b^	67.6 ± 11.6 ^a^	58.0 ± 11.2 ^b^	58.0 ± 6.3 ^b^
ALT (IU/L)	25.8 ± 3.6 ^b^	30.9 ± 6.5 ^a^	24.6 ± 6.8 ^b^	24.5 ± 4.5 ^b^
γ-GT (IU/L)	1.5 ± 0.7 ^b^	2.6 ± 0.1 ^a^	1.6 ± 0.6 ^b^	1.7 ± 0.5 ^b^
TGs (mg/dL)	76.0 ± 16.9 ^b^	97.9 ± 18.9 ^a^	102.8 ± 21.6 ^a^	90.4 ± 16.5 ^ab^
Endotoxin (EU/mL)	0.18 ± 0.05 ^b^	0.28 ± 0.09 ^a^	0.15 ± 0.09 ^b^	0.06 ± 0.03 ^c^
Liver				
Liver index (%)	3.8 ± 0.2 ^c^	4.4 ± 0.2 ^a^	4.0 ± 0.2 ^bc^	4.0 ± 0.4 ^b^
TGs (μmol/liver)	36.0 ± 9.1 ^c^	62.5 ± 18.7 ^a^	50.6 ± 9.1 ^b^	49.6 ± 6.9 ^b^
TNF-α (pg/mg protein)	130.4 ± 35.9 ^b^	155.1 ± 20.8 ^a^	111.4 ± 11.0 ^b^	125.8 ± 9.8 ^b^
IL-1β (pg/mg protein)	129.4± 21.9 ^b^	161.5 ± 12.4 ^a^	113.9 ± 35.0 ^b^	119.2 ± 12.0 ^b^

Values are expressed as mean ± SD (*n* = 10). ^abc^ Significant difference (*p* < 0.05) is identified by different letters in the same row. C, control group; E, ethanol group; E + LP, low-dose pitaya peel extract (PPE) group; E + HP, high-dose PPE group. AST, aspartate transaminase; ALT, alanine transaminase; γ-GT, gamma glutamyl transpeptidase; TGs, triglycerides. Liver index (%) = liver weight/body weight.

## References

[1] World Health Organization (2014). Global Status Report on Alcohol and Health 2014.

[2] Seitz H.K., Bataller R., Cortez-Pinto H., Gao B., Gual A., Lackner C., Mathurin P., Mueller S., Szabo G., Tsukamoto H. (2018). Alcoholic liver disease. Nat. Rev. Dis. Primers.

[3] Kim M.S., Ong M., Qu X. (2016). Optimal management for alcoholic liver disease: Conventional medications, natural therapy or combination?. World J. Gastroenterol..

[4] You M., Matsumoto M., Pacold C.M., Cho W.K., Crabb D.W. (2004). The role of AMP-activated protein kinase in the action of ethanol in the liver. Gastroenterology.

[5] Liu J. (2014). Ethanol and liver: Recent insights into the mechanisms of ethanol-induced fatty liver. World J. Gastroenterol..

[6] Yu S., Rao S., Reddy J.K. (2003). Peroxisome proliferator-activated receptors, fatty acid oxidation, steatohepatitis and hepatocarcinogenesis. Curr. Mol. Med..

[7] Lu Y., Cederbaum A.I. (2008). CYP2E1 and oxidative liver injury by alcohol. Free Radic. Biol. Med..

[8] Bharrhan S., Koul A., Chopra K., Rishi P. (2011). Catechin suppresses an array of signalling molecules and modulates alcohol-induced endotoxin mediated liver injury in a rat model. PLoS ONE.

[9] Glaser T., Baiocchi L., Zhou T., Francis H., Lenci I., Grassi G., Kennedy L., Liangpunsakul S., Glaser S., Alpini G. (2020). Pro-inflammatory signalling and gut-liver axis in non-alcoholic and alcoholic steatohepatitis: Differences and similarities along the path. J. Cell. Mol. Med..

[10] Szabo G. (2015). Gut-liver axis in alcoholic liver disease. Gastroenterology.

[11] Patel S., Behara R., Swanson G.R., Forsyth C.B., Voigt R.M., Keshavarzian A. (2015). Alcohol and the Intestine. Biomolecules.

[12] Rodriguez E.B., Vidallon M.L.P., Mendoza D.J.R., Reyes C.T. (2016). Health-promoting bioactivities of betalains from red dragon fruit (Hylocereus polyrhizus (Weber) Britton and Rose) peels as affected by carbohydrate encapsulation. J. Sci. Food Agric..

[13] Ramli N.S., Brown L., Ismail P., Rahmat A. (2014). Effects of red pitaya juice supplementation on cardiovascular and hepatic changes in high-carbohydrate, high-fat diet-induced metabolic syndrome rats. BMC Complement. Altern. Med..

[14] Suh D.H., Lee S., Heo do Y., Kim Y.S., Cho S.K., Lee S., Lee C.H. (2014). Metabolite profiling of red and white pitayas (Hylocereus polyrhizus and Hylocereus undatus) for comparing betalain biosynthesis and antioxidant activity. J. Agric. Food Chem.

[15] da Silva D.V.T., Dos Santos Baiao D., de Oliveira Silva F., Alves G., Perrone D., Del Aguila E.M., Paschoalin V.M.F. (2019). Betanin, a natural food additive: Stability, bioavailability, antioxidant and preservative ability assessments. Molecules.

[16] Song H., Chu Q., Xu D., Xu Y., Zheng X. (2016). Purified betacyanins from hylocereus undatus peel ameliorate obesity and insulin resistance in high-fat-diet-fed mice. J. Agric. Food Chem..

[17] Lee J.H., Son C.W., Kim M.Y., Kim M.H., Kim H.R., Kwak E.S., Kim S., Kim M.R. (2009). Red beet (*Beta vulgaris* L.) leaf supplementation improves antioxidant status in C57BL/6J mice fed high fat high cholesterol diet. Nutr. Res. Pract..

[18] Fathordoobady F., Mirhosseini H., Selamat J., Manap M.Y. (2016). Effect of solvent type and ratio on betacyanins and antioxidant activity of extracts from Hylocereus polyrhizus flesh and peel by supercritical fluid extraction and solvent extraction. Food Chem..

[19] George S., Brat P., Alter P., Amiot M.J. (2005). Rapid determination of polyphenols and vitamin C in plant-derived products. J. Agric. Food Chem..

[20] Cai Y., Sun M., Wu H., Huang R., Corke H. (1998). Characterization and quantification of betacyanin pigments from diverse Amaranthus species. J. Agric. Food Chem..

[21] Lieber C.S., Decarli L.M. (1994). Animal models of chronic ethanol toxicity. Methods Enzym..

[22] Folch J., Lees M., Sloane Stanley G.H. (1957). A simple method for the isolation and purification of total lipides from animal tissues. J. Biol. Chem..

[23] Yagi K. (1998). Simple assay for the level of total lipid peroxides in serum or plasma. Methods Mol. Biol..

[24] Kleiner D.E., Brunt E.M., Van Natta M., Behling C., Contos M.J., Cummings O.W., Ferrell L.D., Liu Y.C., Torbenson M.S., Unalp-Arida A. (2005). Design and validation of a histological scoring system for nonalcoholic fatty liver disease. Hepatology.

[25] Lieber C.S. (2004). Alcoholic fatty liver: Its pathogenesis and mechanism of progression to inflammation and fibrosis. Alcohol.

[26] Gao F., Zheng K., Benedé-Ubieto R., Cubero F.J., Nevzorova Y.A. (2018). The Lieber-DeCarli diet-a flagship model for experimental alcoholic liver disease. Alcohol Clin. Exp. Res..

[27] Gao B., Bataller R. (2011). Alcoholic liver disease: Pathogenesis and new therapeutic targets. Gastroenterology.

[28] Song H., Chu Q., Yan F., Yang Y., Han W., Zheng X. (2016). Red pitaya betacyanins protects from diet-induced obesity, liver steatosis and insulin resistance in association with modulation of gut microbiota in mice. J. Gastroenterol. Hepatol..

[29] Yin H.Q., Kim M., Kim J.H., Kong G., Kang K.S., Kim H.L., Yoon B.I., Lee M.O., Lee B.H. (2007). Differential gene expression and lipid metabolism in fatty liver induced by acute ethanol treatment in mice. Toxicol. Appl. Pharm..

[30] Fischer M., You M., Matsumoto M., Crabb D.W. (2003). Peroxisome proliferator-activated receptor alpha (PPARalpha) agonist treatment reverses PPARalpha dysfunction and abnormalities in hepatic lipid metabolism in ethanol-fed mice. J. Biol. Chem..

[31] Shklyaev S., Aslanidi G., Tennant M., Prima V., Kohlbrenner E., Kroutov V., Campbell-Thompson M., Crawford J., Shek E.W., Scarpace P.J. (2003). Sustained peripheral expression of transgene adiponectin offsets the development of diet-induced obesity in rats. Proc. Natl. Acad. Sci. USA.

[32] Galli A., Pinaire J., Fischer M., Dorris R., Crabb D.W. (2001). The transcriptional and DNA binding activity of peroxisome proliferator-activated receptor alpha is inhibited by ethanol metabolism. A novel mechanism for the development of ethanol-induced fatty liver. J. Biol. Chem..

[33] Zhang X., Li S.Y., Brown R.A., Ren J. (2004). Ethanol and acetaldehyde in alcoholic cardiomyopathy: From bad to ugly en route to oxidative stress. Alcohol.

[34] Zhao N., Guo F.F., Xie K.Q., Zeng T. (2018). Targeting Nrf2 is a promising intervention approach for the prevention of ethanol-induced liver disease. Cell. Mol. Life Sci..

[35] Lu C., Xu W., Shao J., Zhang F., Chen A., Zheng S. (2017). Nrf2 activation is required for ligustrazine to inhibit hepatic steatosis in alcohol-preferring mice and hepatocytes. Toxicol. Sci..

[36] Mostafavi-Pour Z., Ramezani F., Keshavarzi F., Samadi N. (2017). The role of quercetin and vitamin C in Nrf2-dependent oxidative stress production in breast cancer cells. Oncol. Lett..

[37] Sun J., Fu J., Li L., Chen C., Wang H., Hou Y., Xu Y., Pi J. (2018). Nrf2 in alcoholic liver disease. Toxicol. Appl. Pharmacol..

[38] Tenore G.C., Novellino E., Basile A. (2012). Nutraceutical potential and antioxidant benefits of red pitaya (Hylocereus polyrhizus) extracts. J. Funct. Foods.

[39] Esatbeyoglu T., Wagner A.E., Motafakkerazad R., Nakajima Y., Matsugo S., Rimbach G. (2014). Free radical scavenging and antioxidant activity of betanin: Electron spin resonance spectroscopy studies and studies in cultured cells. Food Chem. Toxicol..

[40] Han J., Zhang Z., Yang S., Wang J., Yang X., Tan D. (2014). Betanin attenuates paraquat-induced liver toxicity through a mitochondrial pathway. Food Chem. Toxicol..

[41] Hritz I., Mandrekar P., Velayudham A., Catalano D., Dolganiuc A., Kodys K., Kurt-Jones E., Szabo G. (2008). The critical role of toll-like receptor (TLR) 4 in alcoholic liver disease is independent of the common TLR adapter MyD88. Hepatology.

[42] Choi Y., Abdelmegeed M.A., Song B.J. (2018). Preventive effects of indole-3-carbinol against alcohol-induced liver injury in mice via antioxidant, anti-inflammatory, and anti-apoptotic mechanism: Role of gut-liver-adipose tissue axis. J. Nutr. Biochem..

[43] Ramli N.S., Ismail P., Rahmat A. (2016). Red pitaya juice supplementation ameliorates energy balance homeostasis by modulating obesity-related genes in high-carbohydrate, high-fat diet-induced metabolic syndrome rats. BMC Complement. Altern. Med..

